# Responses of Phosphate-Solubilizing Microorganisms Mediated Phosphorus Cycling to Drought-Flood Abrupt Alternation in Summer Maize Field Soil

**DOI:** 10.3389/fmicb.2021.768921

**Published:** 2022-01-13

**Authors:** Wuxia Bi, Baisha Weng, Denghua Yan, Hao Wang, Mengke Wang, Siying Yan, Lanshu Jing, Tiejun Liu, Wenjuan Chang

**Affiliations:** ^1^State Key Laboratory of Simulation and Regulation of Water Cycle in River Basin, China Institute of Water Resources and Hydropower Research, Beijing, China; ^2^Yinshanbeilu Grassland Eco-Hydrology National Observation and Research Station, China Institute of Water Resources and Hydropower Research, Beijing, China; ^3^College of Resource Environment and Tourism, Capital Normal University, Beijing, China; ^4^College of Hydrology and Water Resources, Hohai University, Nanjing, China; ^5^Collaborative Innovation Center for Grassland Ecological Security (Jointly Supported by the Ministry of Education of China and Inner Mongolia Autonomous Region), Hohhot, China; ^6^College of Hydraulic and Environmental Engineering, China Three Gorges University, Yichang, China

**Keywords:** drought-flood abrupt alternation, phosphorus cycling, phosphate-solubilizing bacteria, metagenome, farmland ecosystem network

## Abstract

Soil microbial communities are essential to phosphorus (P) cycling, especially in the process of insoluble phosphorus solubilization for plant P uptake. Phosphate-solubilizing microorganisms (PSM) are the dominant driving forces. The PSM mediated soil P cycling is easily affected by water condition changes due to extreme hydrological events. Previous studies basically focused on the effects of droughts, floods, or drying-rewetting on P cycling, while few focused on drought-flood abrupt alternation (DFAA), especially through microbial activities. This study explored the DFAA effects on P cycling mediated by PSM and P metabolism-related genes in summer maize field soil. Field control experiments were conducted to simulate two levels of DFAA (light drought-moderate flood, moderate drought-moderate flood) during two summer maize growing periods (seeding-jointing stage, tasseling-grain filling stage). Results showed that the relative abundance of phosphate-solubilizing bacteria (PSB) and phosphate-solubilizing fungi (PSF) increased after DFAA compared to the control system (CS), and PSF has lower resistance but higher resilience to DFAA than PSB. Significant differences can be found on the genera *Pseudomonas*, *Arthrobacter*, and *Penicillium*, and the P metabolism-related gene K21195 under DFAA. The DFAA also led to unstable and dispersed structure of the farmland ecosystem network related to P cycling, with persistent influences until the mature stage of summer maize. This study provides references for understanding the micro process on P cycling under DFAA in topsoil, which could further guide the DFAA regulations.

## Introduction

Phosphorus (P), a vital nutrient required for plant growth (Shi et al., 2021) but a key factor causing water pollution (Glendell et al., 2014; Yan et al., 2019), is naturally concentrated in topsoil layers (Suriyagoda et al., 2014). The soil P cycling is the input, output, migration, and transformation of P element in the soil (Zhao et al., 2021). Among them, the transformation of insoluble P to soluble P is one of the key processes for plant P uptake (Yin et al., 2021). Soil microbial communities are essential to element cycling, regulation of ecosystem productivity, and so on (Hare et al., 2017; Widdig et al., 2020). As dominant driving force in soil P cycling, phosphate-solubilizing microorganisms (PSM) refer to the group of microbial communities which can solubilize insoluble P to soluble P for plant absorption (Meena et al., 2017). Most PSM are phosphate-solubilizing bacteria (PSB), few are phosphate-solubilizing fungi (PSF) and actinomycetes (Zhang J. et al., 2020).

Soil microbial communities are controlled by soil conditions, such as water content, pH, temperature, available organic material, and by the complex interactions among these factors (Wu et al., 2015; Fierer, 2017; Xue et al., 2018). Among them, water content is one major factor (Wang et al., 2019), so the structure and diversity of soil microbial communities are easily affected by the extreme hydrological events, such as droughts and floods. The increasing frequency and magnitude of these events (Amirataee and Montaseri, 2017; Deng et al., 2018) cause soil water variation, which affects microbial activities, which further impacts P cycling. Currently, the uneven precipitation distribution has caused the rapid increase of drought-flood abrupt alternation (DFAA) events, especially in summer and in southern China (Zhang and Li, 2019). Different from dry-wet cycling or drying-rewetting, DFAA denotes persistent drought followed by sudden heavy precipitation at certain levels (Wu et al., 2006). Frequent DFAA events seriously affect crop growth, yield formation, and environment (Lian et al., 2015; Xiong et al., 2018; Gao et al., 2019), while the dry-wet cycling or drying-rewetting are not at all catastrophic and even have overcompensation effects. At present, there are scant studies on the effects of DFAA on soil microbial communities. In addition, the PSM mediated P cycling has been revealed under individual drought events (Preece et al., 2020; Püschel et al., 2021), individual flood events (Bai et al., 2019; Bao et al., 2019), or the drying-rewetting process (Brödlin et al., 2019; Gao et al., 2020), while few on DFAA. What’s more, many studies have addressed the negative impacts of DFAA on agricultural production (Huang et al., 2019; Xiong et al., 2020). Therefore, it is necessary to reveal the responses of PSM mediated P cycling to DFAA in agroecosystems, especially in topsoil layers.

Our previous study reported that PSB in topsoil increased after DFAA, further increased soil available P (AP), especially for the DFAA with moderate drought (Bi et al., 2020b). It is found that the P solubilizing ability and stability of PSF are stronger than those of PSB although accounting for small proportion (Chittora et al., 2020). Thus, we continue studying the DFAA effects on PSB and PSF. Furthermore, the metagenome (microbial environmental genome) analysis has been used to study the relation between microbial communities and their surrounding environment (Markowitz et al., 2015). We applied the metagenome analysis to obtain the keystone genes related to P metabolism with significant difference under DFAA.

The stability of an ecosystem can be evaluated by co-occurrence network analysis, which can reveal the interactions among microbial communities and altered environmental factors by the complexity and differences of network topologies (Ma et al., 2016; Bello et al., 2020). The main environmental factors affecting P cycling are microbially-mediated activities (Upreti et al., 2019), broken soil structure (Cui et al., 2019), soil organic matter mineralization (Merino et al., 2019), and so on. Does DFAA affect the stability of farmland ecosystem related to P cycling? Destroy or improve the stability? These questions need to be explored.

Here, we conducted field experiments to reveal the mechanism of how DFAA affects soil PSM and their effects on soil P cycling. The aim of this study was to test the following hypotheses: (i) PSB and PSF in topsoil increased after DFAA compared with the control system; (ii) DFAA caused the changes of several genes related to P metabolism compared with the control system; and (iii) the DFAA destabilized and dispersed the farmland ecosystem network related to P cycling. In addition, we observed whether the effects of DFAA on P cycling were persistent until the end of growing season of summer maize. The results of this study can help better understand the influence process of DFAA on P cycling through microbial activities in topsoil, and can further provide a scientific reference for the targeted regulation of DFAA.

## Materials and Methods

### Experimental Design

The experiments were conducted at Wudaogou Experimentation Research Station (33°09′N, 117°21′E), Bengbu City, China. The area has warm temperate, humid, monsoon climate, with DFAA events occurring about every 4 years during 1964–2017, which is ideal for studying DFAA events.

The DFAA events can be evaluated by the method considering meteorological and agricultural indicators on daily scale. Detailed determination method and classification standard can be found in Supplementary Material 1. Supplementary Table 1 shows the determination standard of DFAA in the Northern Anhui Plain. According to historical meteorological data, the probability of DFAA events in light drought-moderate flood (LM) and moderate drought-moderate flood (MM) was 23.7%. Based on the frequency analysis, the frequency for LM and MM was *P* = 20% and *P* = 80%, respectively. Thus, the above two DFAA types were quite important in occurrence, and were probably catastrophic. Therefore, the LM and MM were selected for investigating the effects of DFAA on soil P in this study.

We explored the DFAA effects in two summer maize growing periods, i.e., the seeding-jointing stage (SJS) and the tasseling-grain filling stage (TGS). In total, four different combinations with two DFAA assays and two growing periods were designed, i.e., LMsj (LM during the SJS), MMsj (MM during the SJS), LMtg (LM during the TGS), and MMtg (MM during the TGS). In addition, a control system (CS) under natural climatic conditions was set for reference. The setting details for different experimental treatments are presented in Supplementary Table 2. Notably, there were no interventions of natural rainfall during the experimental period because that the DFAA treatments were sheltered under a ventilated shed with an artificial rainfall device to block the entry of external precipitation and simulate consecutive rainless conditions and abrupt precipitation conditions. For this study, the short-term precipitation corresponding to a moderate flood was 130 mm, with artificial rainfall duration of 1.3 h. For the CS treatment, the soil water content was kept in suitable range for crop growth before the precipitation experiment. Each experimental plot had only one outlet with a water collecting device connected for collecting the surface runoff, the small amount of surface water percolated into the soil during no more than 5 mins, thus there was no standing water after artificial rainfall. All experiment conditions were similar to our previous study (Bi et al., 2020b), except the DFAA treatments. Each treatment had three replicates (experimental plots). The initial conditions (soil water content, P addition, and so on) were kept the same for all the experimental plots.

### Soil Microbial Analysis

**FIGURE 1:**
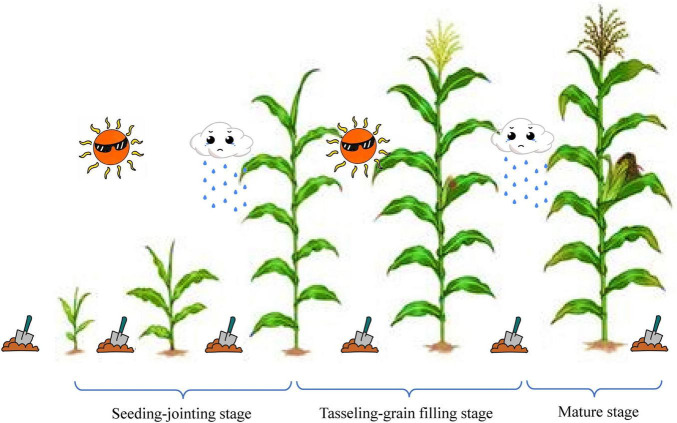
Sampling time points during experiments. DFAA, drought-flood abrupt alternation; BV, baseline value, sampling before the DFAA experiments; BPre, before precipitation, i.e., before drought, sampling 1 day before every rainfall experiment; APre, after precipitation, i.e., after drought, sampling 3 day after every rainfall experiment; M, mature stage, sampling at the mature stage.

The soil samples for microbial analysis were collected on specific days (Figure 1): before the DFAA experiments (BV, i.e., baseline value), 1 day before (BPre, i.e., after drought) and 3 days after (APre, i.e., after DFAA) every artificial rainfall experiment, and at the mature stage (M). 5 g soil sample was collected from topsoil (0–40 cm) for soil microbial analysis. Soil samples were frozen at –80°C until DNA extraction. As our study focuses on PSB and PSF, bacterial and fungal communities in topsoil were measured. Universal primers, specifically 338F (5′-ACTCCTACGGGAGGCAGCAG-3′) and 806R (5′-ACTCCTACGGGAGGCAGCAG-3′), ITS1F (5′-CTTGGTCATTTAGAGGAAGTAA-3′) and ITS2R (5′-GCTGCGTTCTTCATCGATGC-3′), were used for polymerase chain reaction (PCR) amplification of bacterial and fungal communities, respectively. The specific processes of DNA extraction and high-throughput sequencing were described in our previous study (Bi et al., 2020b) and completed by Shanghai Majorbio Bio-Pharm Technology Co. Ltd. (Shanghai, China).

The metagenomics techniques were applied to explore the DFAA effects on P metabolism. E.Z.N.A.^®^ Soil DNA Kit (Omega Bio-tek, United States) was used for DNA extraction. After genomic DNA extraction, TBS-380 was used to detect DNA concentration, NanoDrop2000 was used to detect DNA purity, and then 1% agarose gel electrophoresis was used to detect DNA integrity. The DNA was fragmented by Covaris M220 (Gene Company Limited, China), and fragments of approximately 400 bp were screened. The PE library was constructed by using library construction kit of NEXTFLEX Rapid DNA-Seq (Bioo Scientific, United States). The sequencing was performed on the Illumina NovaSeq/Hiseq Xten (Illumina, United States) platform.

### Soil Physicochemical Analysis

The soil samples for physicochemical analysis were collected on specific days same with that for microbial analysis, except the samples were collected 1 day after every artificial rainfall experiment for APre. For P analysis, the concentrations of AP and total P (TP) were measured according to the *Phosphorus Determination Methods of Forest Soils Standard in China* (LY/T 1232-2015) via the colorimetry method and the alkali fusion method, respectively (Supplementary Material 2). Soil organic matter content (OMC) was analyzed by exothermic heating and oxidation with the potassium dichromate method (Nelson and Sommers, 2018). Soil pH was estimated on a soil/water ratio (w/v) of 1:2.5 (PHS-3C pH acidometer, China) using a glass electrode meter. Soil total porosity (STP) was calculated based on soil bulk density and soil specific gravity, which was determined by the ring cutting method and pycnometer method, respectively (dos Reis et al., 2021).

### Statistical Analysis

Data were shown in mean ± standard deviation. Variables were analyzed with one-way ANOVA. The significance of linear correlations among parameters was expressed as Pearson’s product moment correlation coefficient. A statistical difference was considered significant when *P* < 0.05. All the data processing was performed using SPSS version 20.0 (SPSS Inc., Chicago, IL, United States) and R language (version 3.5.3). The figures were drawn in Origin version 9.0 (OriginLab Inc., Hampton, MA, United States) and CANOCO model (version 4.5). Co-occurrence network analysis was performed using Gephi software (version 0.9.2) based on the Spearman correlation index (*P* < 0.05).

Especially, Kruskal-Wallis H test, and LEFSe (Linear discriminant analysis Effect Size) test were applied for metagenomics data analysis. Kruskal-Wallis H test is a method of significant differences analysis for species in multiple groups, which was performed using R project Vegan package (Sun et al., 2021). LEFSe is a software for discovering high-dimensional biomarkers and revealing genome characteristics among two or more biological groups (LEFSe, 2011 online analysis).

## Results

### Shift in Soil Phosphate-Solubilizing Microorganisms Under Drought-Flood Abrupt Alternation

#### Phosphate-Solubilizing Bacteria

**FIGURE 2:**
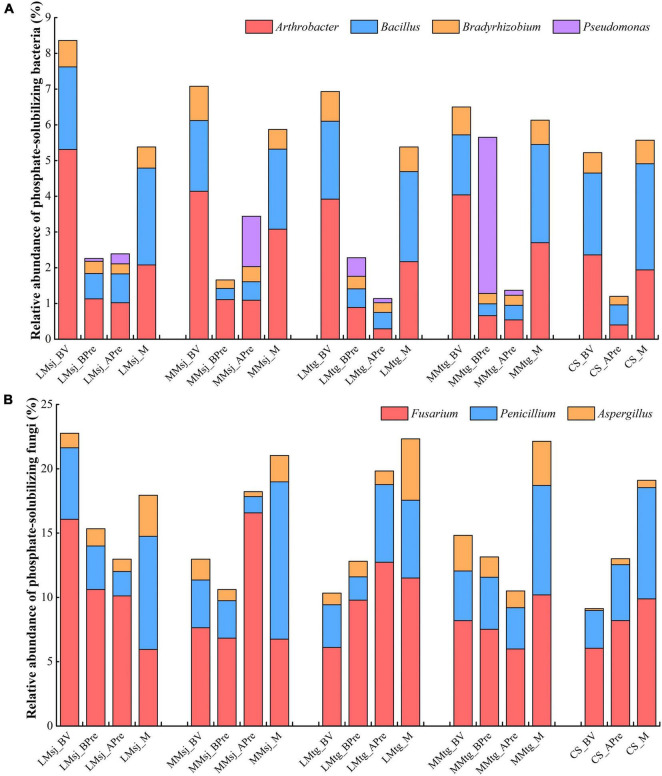
The relative abundance of **(A)** phosphate-solubilizing bacteria. **(B)** Phosphate-solubilizing fungi. LMsj, light drought-moderate flood in the seeding-jointing stage (SJS); LMtg, light drought-moderate flood in the tasseling-grain filling stage (TGS); MMsj, moderate drought-moderate flood in the SJS; MMtg, moderate drought-moderate flood in the TGS; CS, control system operated under natural climatic conditions; BV, baseline value; BPre, before precipitation (after drought); APre, after precipitation (after drought-flood abrupt alternation/natural rainfall); M, at the mature stage.

The main PSB genera found in the samples were *Arthrobacter*, *Bacillus*, *Bradyrhizobium*, and *Pseudomonas* (Figure 2A). The relative abundance of PSB in most treatments were between 1 and 7%. Compared with baseline values (BV), the relative abundance of PSB before DFAA (BPre) decreased 73.0, 76.6, 67.1, and 13.1% in the LMsj, MMsj, LMtg, and MMtg treatments, respectively. In the LMsj, MMsj, LMtg, MMtg, and CS treatments, the relative abundance of PSB after DFAA/natural rainfall (APre) decreased 71.4, 51.4, 83.5, 78.9, and 77.0% than BV, respectively. Compared with the CS treatment, the relative abundance of PSB APre was higher by 99.2, 186.7, −5.0, and 14.2% in the LMsj, MMsj, LMtg, and MMtg treatments. For the mature stage, the relative abundance of PSB in the CS treatment was −3.4, 5.4, −3.4, and 10.1% lower than that in the LMsj, MMsj, LMtg, and MMtg treatments, respectively.

**FIGURE 3:**
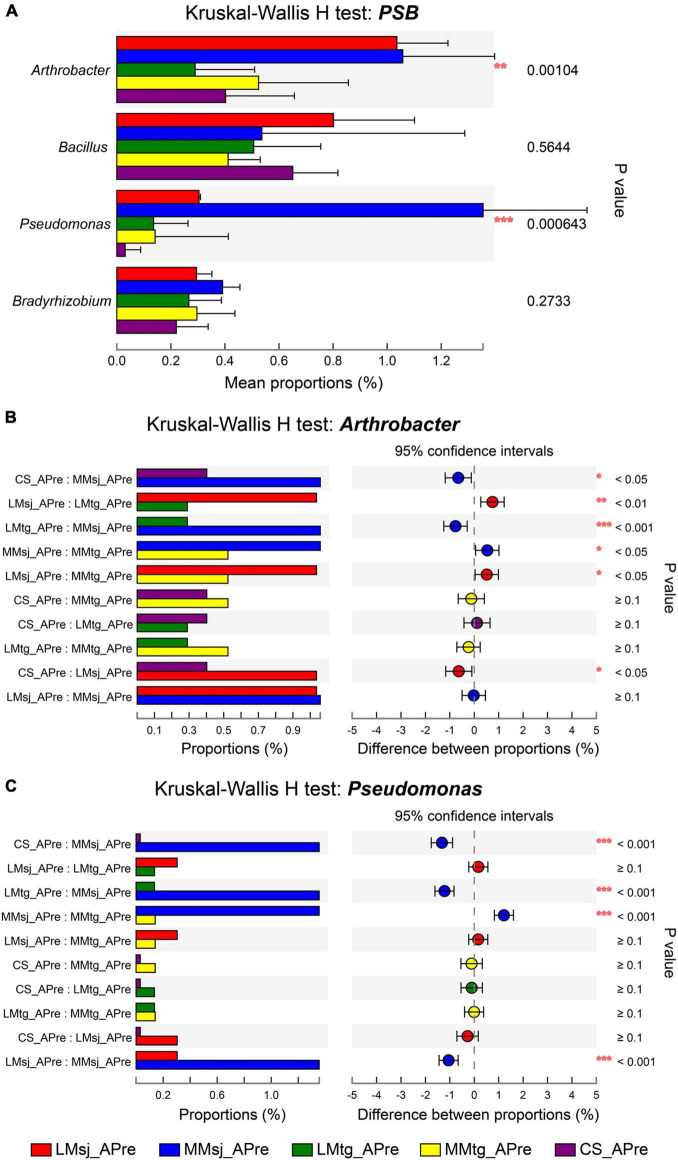
**(A)** The Kruskal-Wallis H test bar plot for phosphate-solubilizing bacteria after drought-flood abrupt alternation (DFAA)/natural rainfall. **(B)** The *post hoc* plot of Kruskal-Wallis H test of genus *Arthrobacter*. **(C)** The *post hoc* plot of Kruskal-Wallis H test of genus *Pseudomonas*. *P < 0.05; **P < 0.01; ***P < 0.001.

The significance of difference for PSB in topsoil under different treatments was further explored. Figure 3A shows the Kruskal-Wallis H test for PSB after DFAA/natural rainfall. The genus with strong significant difference (*P* < 0.001) after DFAA was *Pseudomonas* (*P* = 0.000643). The genus with highly significant difference (*P* < 0.01) was *Arthrobacter* (*P* = 0.00104). To get more details, *post hoc* plots were presented with comparation of every two treatments for genera *Arthrobacter* and *Pseudomonas*. For *Arthrobacter*, there was strong significant difference (*P* < 0.001) between LMtg and MMsj treatments; highly significant difference (*P* < 0.01) was found between LMsj and LMtg treatments; significant difference (*P* < 0.05) was found in four pairs: CS and MMsj treatments, MMsj and MMtg treatments, LMsj and MMtg treatments, CS and LMsj treatments (Figure 3B). For *Pseudomonas*, there was strong significant difference (*P* < 0.001) between: CS and MMsj treatments, LMtg and MMsj treatments, MMsj and MMtg treatments, LMsj and MMsj treatments (Figure 3C). The Kruskal-Wallis H test of PSB at the mature stage was presented in Supplementary Figure 1. For the four PSB genera, there were no significant difference (*P* > 0.05) among DFAA treatments and CS treatment.

#### Phosphate-Solubilizing Fungi

*Fusarium*, *Penicillium*, and *Aspergillus* were the main PSF genera in topsoil (Figure 2B). The relative abundance of PSF were between 10 and 25%. In the LMsj, MMsj, LMtg, and MMtg treatments, the relative abundance of PSF before DFAA (BPre) decreased by 32.6, 18.2, −24.0, and 11.3% than BV, respectively. Compared with BV, the relative abundance of PSF after DFAA/natural rainfall in the LMsj, MMsj, LMtg, MMtg, and CS treatment decreased 43.0, −40.5, 91.9, 29.1, and −42.4%, respectively. Compared with the CS treatment, the relative abundance of PSF after DFAA was higher by −0.3, 40.0, 52.4, and −19.3% in the LMsj, MMsj, LMtg, and MMtg treatments. The relative abundance of PSB at the mature stage in the LMsj, MMsj, LMtg, and MMtg treatments was −6.1, 10.1, 16.8, and 15.9% higher than in the CS treatment, respectively.

**FIGURE 4:**
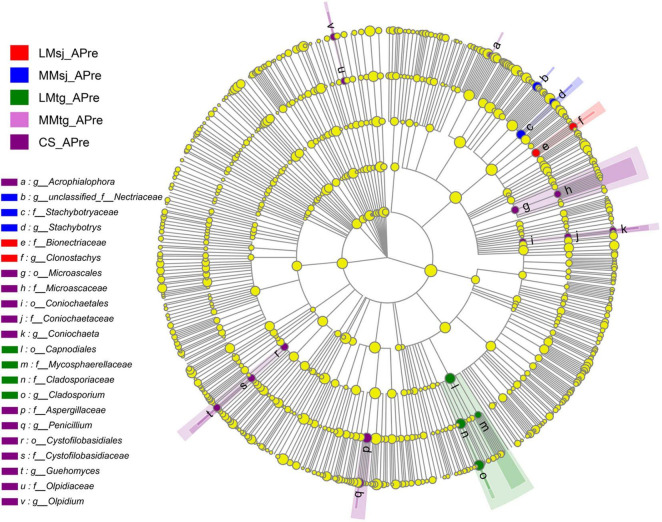
The LEFSe cladogram of fungal communities (containing phosphate-solubilizing fungi) after DFAA/natural rainfall.

Figure 4 plots the LEFSe test result of fungi in topsoil from phylum to genus level APre, which concluded the above-mentioned PSF. Significant differences among the DFAA treatments and CS treatment were found for genus *Penicillium* (dark purple point). At the mature stage, there were no significant difference among the DFAA treatments and CS treatment for the PSF genera (Supplementary Figure 2).

### Shift in Soil Phosphorus Metabolism Metagenomic Under Drought-Flood Abrupt Alternation

The functional genes related to P metabolism were screened in the Kyoto Encyclopedia of Genes and Genomes (KEGG), one of the commonly used international biological information databases. The KEGG Pathway Level 3 ko00440 (phosphonate and phosphinate metabolism) is most related to the P metabolism in soil and crops. Compared with the soil microbial samples in this study, 15 KEGG Orthology (KO) were found. The specific homologous gene numbers, function descriptions and EC numbers were shown in Table 1.

**TABLE 1:** The Kyoto Encyclopedia of Genes and Genomes Orthology (KEGG) Orthology related to phosphorus metabolism in topsoil layer (0–40 cm).

KO	Function description	EC
K01841	Phosphoenolpyruvate phosphomutase	5.4.2.9
K03430	2-Aminoethylphosphonate-pyruvate transaminase	2.6.1.37
K03823	Phosphinothricin acetyltransferase	2.3.1.183
K05306	Phosphonoacetaldehyde hydrolase	3.11.1.1
K06162	Alpha-D-ribose 1-methylphosphonate 5-triphosphate diphosphatase	3.6.1.63
K06163	Alpha-D-ribose 1-methylphosphonate 5-phosphate C-P lyase	4.7.1.1
K06164	Alpha-D-ribose 1-methylphosphonate 5-triphosphate synthase subunit PhnI	2.7.8.37
K06165	Alpha-D-ribose 1-methylphosphonate 5-triphosphate synthase subunit PhnH	2.7.8.37
K06166	Alpha-D-ribose 1-methylphosphonate 5-triphosphate synthase subunit PhnG	2.7.8.37
K06167	Phosphoribosyl 1,2-cyclic phosphate phosphodiesterase	3.1.4.55
K09459	Phosphonopyruvate decarboxylase	4.1.1.82
K12909	Carboxyvinyl-carboxyphosphonate phosphorylmutase	2.7.8.23
K19670	Phosphonoacetate hydrolase	3.11.1.2
K21195	2-Aminoethylphosphonate dioxygenase	1.14.11.46
K21196	2-Amino-1-hydroxyethylphosphonate dioxygenase (glycine-forming)	1.13.11.78

**FIGURE 5:**
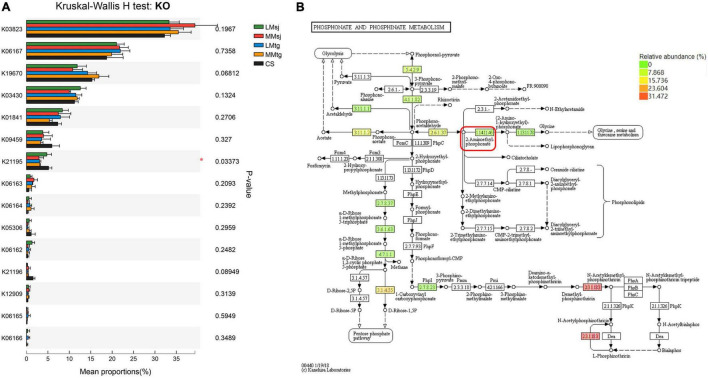
**(A)** The Kruskal-Wallis H test bar plot of Kyoto Encyclopedia of Genes and Genomes Orthology (KO) relating to phosphorus metabolism in topsoil; **(B)** The diagram of phosphorus metabolism pathway in topsoil. *P < 0.05.

The Kruskal-Wallis H test was conducted to explore the significance of differences on the KO related to P metabolism in topsoil after DFAA (Figure 5A). Among all 15 KO, significant difference (*P* < 0.05) was found among DFAA treatments and CS treatment in K21195 (*P* = 0.03373). Combining with the diagram of P metabolism pathway in topsoil, it can be learned that the corresponding EC code of K21195 was 1.14.11.46, with function of 2-aminoethylphosphonate dioxygenase (Figure 5B).

### Variation in Soil Phosphorus and Related Physicochemical Properties Under Drought-Flood Abrupt Alternation

#### Soil Phosphorus

The concentrations of AP for the BV, APre, and at the M in the DFAA treatments were 31.9, 47.5, and 45.5% lower than in the CS treatment, respectively (Table 2). Compared with BV, the AP concentrations BPre, APre, and at the M decreased by 20.4, 28.2, and 32.4% in the DFAA treatments, respectively. While for the CS treatment, the AP concentrations APre and at the M was 6.8 and 15.4% lower than BV. The concentrations of AP in the DFAA treatments with moderate drought was −5.6, 10.9, 28.1, and 37.1% higher than with light drought (with the same flood level) in BV, BPre, APre, and M (17.6% on average). For the DFAA with moderate flood, the AP concentration in the DFAA treatments with moderate drought was higher than that of light drought. The AP concentrations were 22.3, −7.7, 26.7, and 2.8% greater in the tasseling-grain filling stage than in the seeding-jointing stage for BV, BPre, APre, and M (11.0% on average).

**TABLE 2:** Concentration of available phosphorus (AP) and total phosphorus (TP) in topsoil (0–40 cm).

Parameter	Treatment	BV	BPre	APre	M
AP (mg kg^–1^)	LMsj	36.88 ± 3.58^b^	27.94 ± 2.60^b^	22.19 ± 2.97^c^	20.60 ± 1.84^c^
	MMsj	29.22 ± 2.69^c^	32.88 ± 1.73^a^	24.33 ± 1.74^c^	28.39 ± 0.44^b^
	LMtg	38.73 ± 3.86^b^	27.51 ± 1.68^b^	24.04 ± 1.64^c^	21.30 ± 1.56^c^
	MMtg	42.13 ± 5.92^b^	28.61 ± 1.68^b^	34.91 ± 1.89^b^	29.04 ± 2.28^b^
	CS	53.91 ± 2.10^a^	–	50.25 ± 3.04^a^	45.60 ± 2.40^a^
TP (%)	LMsj	0.047 ± 0.0032^a^	0.045 ± 0.0019^a^	0.038 ± 0.0021^d^	0.038 ± 0.0014^d^
	MMsj	0.046 ± 0.0024^a^	0.044 ± 0.0017^a^	0.040 ± 0.0027^d^	0.039 ± 0.0018^cd^
	LMtg	0.053 ± 0.0029^a^	0.042 ± 0.0015^a^	0.045 ± 0.0015^c^	0.041 ± 0.0018^c^
	MMtg	0.053 ± 0.0051^a^	0.044 ± 0.0013^a^	0.049 ± 0.0026^b^	0.044 ± 0.0023^b^
	CS	0.057 ± 0.0038^a^	–	0.055 ± 0.0026^a^	0.050 ± 0.0023^a^
AP/TP (%)	LMsj	7.88	6.26	5.79	5.49
	MMsj	6.31	7.56	6.10	7.23
	LMtg	7.29	6.48	5.38	5.23
	MMtg	7.94	6.56	7.10	6.54
	CS	9.40	–	9.11	9.06

*Data were shown in mean ± standard deviation, the superscripted letters were the analysis results of statistical difference (P < 0.05).*

For TP, the concentrations for BV, APre, and M were 13.1, 22.0, and 19.6% lower in the DFAA treatments than in the CS treatments, respectively (Table 2). The TP concentrations after drought, after DFAA, and at the mature stage decreased by 12.6, 13.6, and 18.8% than BV in the DFAA treatments. While for the CS treatment, the concentrations of TP after DFAA/natural rainfall and at the mature stage was 3.8 and 12.2% lower than BV, respectively. The concentrations of TP in the DFAA treatments with moderate drought was −0.6, 0.0, 7.3, and 7.0% higher than with light drought (with the same flood level) in BV, BPre, APre, and M (3.4% on average). The TP concentrations after DFAA were 14.1, −2.4, 20.0, and 10.9% greater in the tasseling-grain filling stage than in the seeding-jointing stage (10.7% on average).

The ratio of AP to TP for BV, APre, and M were 21.7, 33.1, and 32.4% lower in the DFAA treatments than in the CS treatments, respectively (Table 2). The ratio for BPre, APre, and M decreased by 6.8, 13.4, and 13.1% than BV in the DFAA treatments. While for the CS treatment, the ratio for APre and M was 3.1 and 3.7% lower than BV, respectively. The ratio in the DFAA treatments with moderate drought was −6.1, 10.8, 18.1, and 28.4% higher than with light drought (with the same flood level) in BV, BPre, APre, and M (12.8% on average). The ratio after DFAA were 7.3, −5.6, 5.0, and −7.5% greater in the tasseling-grain filling stage than in the seeding-jointing stage (−0.2% on average).

#### Related Physicochemical Properties

The STP in topsoil increased in the order of BV < BPre < APre < M (Table 3). The STP after DFAA was higher in the treatments with moderate drought (MMsj and MMtg treatments) compared to those with light drought (LMsj and LMtg treatments), by 7.8 and 13.3% in the seeding-jointing stage and in the tasseling-grain filling stage, respectively. The STP APre were higher by 7.1% in the DFAA treatments than in the CS treatment. The STP after drought was slightly higher (10.9%) in the treatments with moderate drought compared to those with light drought. The STP in BV, BPre, APre, and M was 20.2, 26.7, 32.4, and 32.7% greater in the tasseling-grain filling stage than in the seeding-jointing stage.

**TABLE 3:** Concentration of physicochemical parameters in topsoil (0–40 cm).

Parameter	Treatment	BV	BPre	APre	M
Soil total porosity (%)	LMsj	33.31 ± 2.24^c^	33.95 ± 3.72^b^	37.79 ± 2.98^d^	47.24 ± 2.30^b^
	MMsj	34.21 ± 0.77^c^	36.74 ± 0.74^b^	40.75 ± 0.44^cd^	39.66 ± 2.25^c^
	LMtg	42.74 ± 3.89^a^	43.54 ± 2.70^a^	48.71 ± 2.20^b^	56.90 ± 2.95^a^
	MMtg	38.43 ± 0.94^b^	46.06 ± 2.55^a^	55.20 ± 2.75^a^	58.42 ± 3.00^a^
	CS	33.15 ± 1.66^c^	–	42.59 ± 3.00^c^	60.55 ± 3.25^a^
pH	LMsj	7.76 ± 0.041^a^	7.31 ± 0.023^a^	7.42 ± 0.019^a^	7.63 ± 0.12^a^
	MMsj	7.79 ± 0.055^a^	7.38 ± 0.051^a^	7.42 ± 0.13^a^	7.54 ± 0.16^a^
	LMtg	7.78 ± 0.055^a^	7.49 ± 0.14^a^	7.52 ± 0.13^a^	7.57 ± 0.17^a^
	MMtg	7.64 ± 0.035^b^	7.48 ± 0.12^a^	7.52 ± 0.13^a^	7.54 ± 0.15^a^
	CS	6.98 ± 0.018^c^	–	6.71 ± 0.14^b^	7.20 ± 0.18^b^
Organic matter content (g kg^–1^)	LMsj	9.72 ± 0.23^b^	10.66 ± 0.37^a^	8.70 ± 0.51^d^	10.97 ± 0.85^b^
	MMsj	9.24 ± 0.51^b^	11.96 ± 1.26^a^	9.66 ± 1.45^cd^	11.05 ± 1.05^b^
	LMtg	11.09 ± 1.53^ab^	11.70 ± 1.30^a^	11.59 ± 1.28^bc^	10.45 ± 1.50^b^
	MMtg	12.18 ± 1.79^a^	11.44 ± 1.40^a^	13.11 ± 1.10^ab^	12.05 ± 1.45^b^
	CS	12.89 ± 0.83^a^	–	14.81 ± 1.90^a^	14.80 ± 1.90^a^

*Data were shown in mean ± standard deviation, the superscripted letters were the analysis results of statistical difference (P < 0.05).*

The pH after DFAA in the DFAA treatments was 0.77 (11.4%) higher than in the CS treatments (Table 3). There were no significant differences in pH among the DFAA treatments with moderate drought or with light drought (with the same flood level). The pH in BV, BPre, APre, and M was −0.8, 1.9, 1.3, and −0.4% greater in the tasseling-grain filling stage than in the seeding-jointing stage.

The OMC after DFAA in the DFAA treatments was 4.04 g kg^–1^ (27.3%) lower than that in the CS treatments (Table 3). The OMC in the DFAA treatments with moderate drought was 12.2% higher than with light drought (with the same flood level). For BV, BPre, APre, and M, the OMC was 22.7, 2.4, 34.5, and 2.2% greater in the tasseling-grain filling stage than that in the seeding-jointing stage.

### Relationship Among Soil Phosphorus, Related Physicochemical Properties, and Phosphate-Solubilizing Microorganisms

**FIGURE 6:**
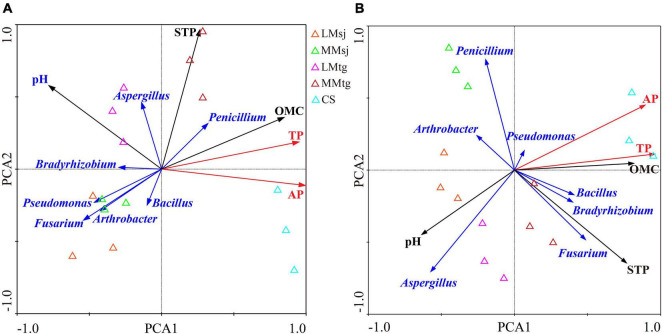
The principal components analysis (PCA) of topsoil parameters related to phosphorus cycle **(A)** after DFAA/natural rainfall; **(B)** at the mature stage.

Distinct separations were observed for soil parameters related to P cycle among different treatments along with axis1 and axis2 of the principal components analysis (PCA) both APre and at the M, especially among the four DFAA treatments and CS treatment (Figure 6). For APre, the eigenvalue of the PCA first axis and the second axis was 0.736 and 0.258, respectively. The STP, OMC, pH, and *Fusarium* in topsoil had significant impacts on AP and TP, with long arrow length. *Aspergillus*, *Penicillium*, *Arthrobacter*, and *Pseudomonas* had moderate impacts on AP and TP, with the arrow length decreasing. *Bradyrhizobium* and *Bacillus* had weak impacts on AP and TP, with the smallest arrow length. The STP, OMC, and *Penicillium* were positively correlated with AP/TP (Figure 6A). For M, the eigenvalue of the PCA first axis and the second axis was 0.700 and 0.291, respectively. *Aspergillus*, STP, pH, *Penicillium*, and OMC in topsoil had significant impacts on AP and TP, with long arrow length. *Fusarium*, *Bradyrhizobium*, and *Bacillus* had moderate impacts on AP and TP, with the arrow length decreasing. *Arthrobacter* and *Pseudomonas* had weak impacts on AP and TP, with the smallest arrow length. Positive correlations were found among OMC, STP, *Pseudomonas*, *Bradyrhizobium*, *Bacillus*, *Fusarium*, and AP/TP (Figure 6B).

**FIGURE 7:**
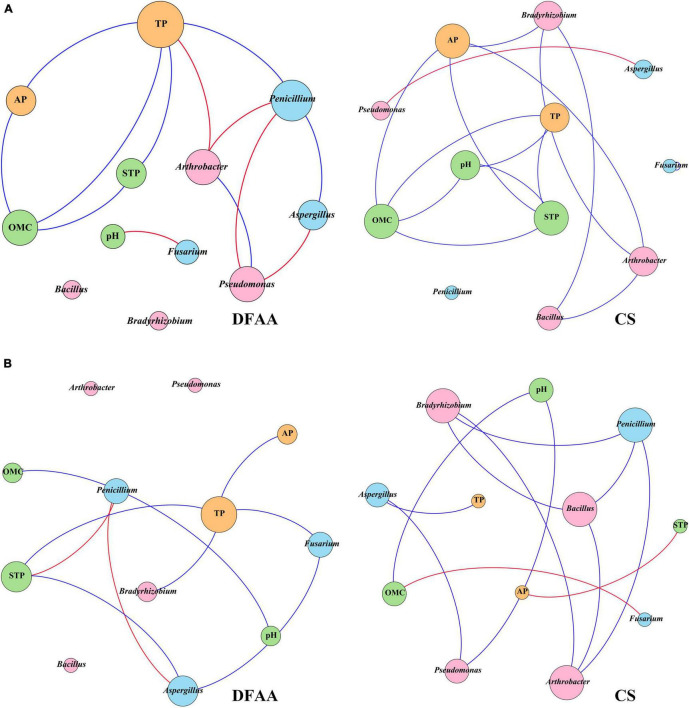
The co-occurrence networks between phosphate-solubilizing microorganisms and soil physicochemical properties related to phosphorus metabolism **(A)** after DFAA/natural rainfall (APre); **(B)** at the mature period (M). Blue lines represent positive correlations, red lines represent negative correlations (Spearman’s | ρ| > 0.6, *P* < 0.05). Orange nodes represent the phosphorus in different forms, pink nodes represent the phosphate-solubilizing bacteria, light blue nodes represent the phosphate-solubilizing fungi, green nodes represent the soil physicochemical parameters related to phosphorus metabolism.

Co-occurrence network analysis (Spearman’s | ρ| > 0.6, *P* < 0.05) was performed between PSM and physicochemical properties related to P in different treatments (Figure 7). Table 4 shows the topological properties of co-occurrence networks. After DFAA/natural rainfall, the modularity index (Mod) values in the DFAA treatments and CS treatment were 0.359 and 0.379, respectively. At the mature stage, the Mod were 0.479 and 0.437 in the DFAA treatments and CS treatment, respectively. The edges/nodes after DFAA were 1.3 and 1.4 for the DFAA treatments and CS treatment, while both were 1.0 at the mature stage. The average clustering coefficient (avgCC) and average path length (APL) were 0.725 and 1.759 in the DFAA treatments after DFAA, 0.75 and 1.862 in the CS treatments after DFAA, 0.333 and 1.759 in the DFAA treatments at the mature stage, 0.5 and 1.909 in the CS treatments at the mature stage, respectively.

**TABLE 4:** Topological properties of co-occurrence networks.

Topological properties	Figure 7A		Figure 7B	
	DFAA	CS	DFAA	CS
Number of nodes	10	10	9	12
Number of edges	13	14	9	12
Ratio of positive edges (%)	61.538	92.857	77.778	83.333
Ratio of negative edges (%)	38.462	7.143	22.222	16.667
Average degree (avgK)	2.6	2.8	2	2
Network diameter	3	4	3	5
Graph density	0.289	0.311	0.25	0.182
Modularity (Mod)	0.359	0.379	0.479	0.437
Average clustering coefficient (avgCC)	0.725	0.75	0.333	0.5
Average path length (APL)	1.759	1.862	1.759	1.909

## Discussion

### The Phosphate-Solubilizing Fungi Were Less Resistant but More Resilient Than Phosphate-Solubilizing Bacteria to Drought-Flood Abrupt Alternation

Immobilization, transformation, and fractionation of soil P can be affected by PSM (Katznelson et al., 1962), which mainly contains PSB, PSF, and Actinomycetes (Rafi et al., 2019). Our study mainly focused on PSB and PSF in topsoil (0–40 cm), where P is naturally concentrated and can easily be affected by environmental changes (Suriyagoda et al., 2014; Missong et al., 2018). The results show that *Arthrobacter* (a type of Actinobacteria), *Bacillus* (a type of Firmicutes), *Bradyrhizobium* (a type of Proteobacteria), and *Pseudomonas* (a type of Proteobacteria) were the four main PSB genera. It is reported that *Bacillus*, *Arthrobacter*, and *Azotobacter* genera appeared more at maize rhizosphere (Cavaglieri et al., 2009; Anzuay et al., 2017), Proteobacteria and Actinobacteria mainly contains the genes that can possibly enhance polyphosphate transformation and the availability of inorganic P in the maize rhizosphere (Li et al., 2014; Wen et al., 2016). Our study proved previous findings. As for PSF, except most studied genera *Penicillium* and *Aspergillus* (Yin et al., 2015; Qiao, 2019), *Fusarium* was also found in topsoil samples in our study.

Compared with the CS treatment, the relative abundance of PSB and PSF after DFAA were higher by 0.89 and 2.37% on average in the DFAA treatments, while by 2.00 and 6.09% in BV. Thus, the relative abundance of PSB and PSF relatively decreased although the absolute value after DFAA increased. And the PSB was more resistant than the PSF. Compared with BV, the PSB after DFAA decreased 71.3 and 77.0% in the DFAA treatments and CS treatment, while they were −20.2 and 6.7% higher at the mature stage in the DFAA treatments and CS treatment, respectively. The PSF after DFAA were 15.1 and 42.4% lower than BV in the DFAA treatments and CS treatment, while they were 51.6 and 109.1% higher at the mature stage than BV in the DFAA treatments and CS treatment, respectively. It is indicated that DFAA can possibly relieve the PSB decrease after flood (precipitation) as the decrease proportion in DFAA treatments were lower than in the CS treatment. The PSB proportion cannot be recovered to the initial state after DFAA as the relative abundance of PSB at the mature stage was still lower than BV in the DFAA treatments while was higher in the CS treatment. The PSF proportion recovered to the initial state as the relative abundance of PSF at the mature stage was higher than BV, while the resilience was weaker than the CS treatment. Therefore, the PSF has higher resilience than PSB under DFAA.

In total, the PSF were less resistant but more resilient than PSB to DFAA. Previous studies have suggested that fungal communities are more sensitive than bacterial communities to changing soil moisture (McHugh and Schwartz, 2016; Jiao et al., 2021). Generally, soil bacteria have lower resistance but higher resilience to drought than fungi (de Vries and Shade, 2013; de Vries et al., 2018). It has also been reported that soil microbial communities are resistant and resilient to climatic extremes, i.e., regular, seasonal fluctuations in temperature and rainfall experienced by such ecosystems (Cruz-Martínez et al., 2009; Kaurin et al., 2018). Drying–rewetting decreased bacterial growth while fungal growth remained unaffected (Bapiri et al., 2010). In details, bacterial community to dry-down and wet-up, while the fungal community was largely unaffected by dry-down, showing marked resistance (Barnard et al., 2013). Our results have findings contrary to that in drought condition, while part was consistent with to that in flood condition (resistance) and in drying-wetting condition (resilience). It indicates that the effects of DFAA on soil microbial communities were not simply the superposition effect of drought and flood, but also had differences with drying-rewetting. The specific effect mechanisms need further exploration.

### Phosphorus Metabolism-Related Gene K21195 Was Significantly Different Under Drought-Flood Abrupt Alternation

The phosphorus metabolism-related metagenomic analysis revealed that significant difference was found among the DFAA treatments and CS treatment in K21195, which mainly affects the synthesis of glycine, serine, threonine and lipophosphoglycan. The glycine, serine and threonine mainly affect the synthesis of protein (Chrystal et al., 2020). The lipophosphoglycan is mainly fixed on the cell membrane through glycosylphosphatidylinositol, which is a multifunctional toxicity determinant (Martinez et al., 2010). It indicates that DFAA will affect protein synthesis and may be toxic to microbial cells. Our previous study reported that DFAA can promote microbial death, the PSB and PSF also decreased after DFAA in this study, which verified the toxic function of K21195.

### The Drought-Flood Abrupt Alternation Decreased Soil Available Phosphorus Transformation While Increased Soil Phosphorus Loss

The AP concentration under DFAA in this study showed same laws with our previous study (Bi et al., 2020b) in the comparisons of different drought levels and summer maize growing periods. The longer duration of droughts, the higher concentration of AP (Zhang H. et al., 2020). The causes of the phenomenon were reported as microbial death (Dijkstra et al., 2015) and soil organic matter decomposition (Yang et al., 2019), which were verified in our study. However, several different results were found between this study and our previous study: the concentrations of AP in the DFAA treatments after DFAA decreased more than in the CS treatment after natural rainfall in this study, while opposite trend was found in our previous study. It is mainly due to the fact that the DFAA treatments were with moderate flood in this study, while were with light flood in previous study and the scouring effect was stronger with moderate flood (Yang et al., 2021). The soil porosity increased significantly in this study, thus, the infiltration effect was strong (Lipiec et al., 2006). These phenomena accelerated the P loss in topsoil. Notably, the absolute relative abundances of PSB and PSF after DFAA in the DFAA treatments were higher than in the CS treatment. While at the same time, the relative abundances of PSB and PSF after DFAA relatively decreased compared with that after natural rainfall in CS treatment. Normally, the AP should relatively increase after DFAA due to PSM which could enhance the formation of AP (Qian et al., 2019). The different results indicated that the P loss effect was stronger than the PSM functions for AP formation in the DFAA treatments with moderate flood. In addition, the P metabolism-related gene K21195 affected protein synthesis and was toxic to microbial cells, which further inhibits the relevant enzyme activities for converting insoluble P into soluble P (including AP). Several studies reported that flooding could increase soil AP because of soil organic matter mineralization (Tang et al., 2016), microbially-mediated activities (Unger et al., 2009), increasing pH (Bai et al., 2017), and soil porosity 378 (Guillaume et al., 2016), the latter two of which were verified in our study. It means that the effects of DFAA on soil P was not the superposition effect of drought and flood, the mechanisms were complicated, depending on the drought level and flood level in DFAA events. The AP concentration in the DFAA treatments at the mature stage recovered a little compared with the CS treatment. The reasons could be that the normal water supply condition was beneficial to the survival of PSM, as the relative abundance of PSB and PSF increased at the mature stage, which further promoted the AP formation. Furthermore, the effect of scouring and infiltration significantly decreased, the soil P loss decreased as well.

### The Drought-Flood Abrupt Alternation Continuously Dispersed the Farmland Ecosystem Network Related to Phosphorus Cycling

Principal components analysis showed that all soil parameters related to P cycling were affected by different treatments both APre and at the mature stage, as there was significant distance among the DFAA treatments and CS treatment (Figure 6). It can be inferred that the DFAA had significant effects on PSM in topsoil, which further affected P cycling, and finally resulted in the yield reduction and water pollution which was found in our previous study (Bi et al., 2020a,b).

The co-occurrence network can be considered as stable when the Mod were larger than 0.4 (Gu et al., 2021). Based on the topological properties of co-occurrence networks (Table 4), the Mod were both smaller than 0.4 after DFAA in the DFAA treatments and after natural rainfall in the CS treatments, while the Mod were larger than 0.4 at the mature stage in all treatments. It can be learned that after DFAA/natural rainfall, the farmland ecosystem network related to P cycling was unstable; while at the mature stage, the farmland ecosystem network related to P cycling restored stability. The higher the modularity, more stable the network structure (Tumiran and Sivakumar, 2021). Thus, the stability of network after DFAA in the DFAA treatments (Mod = 0.359) was weaker than after natural rainfall in the CS treatment (Mod = 0.379). Interestingly, at the mature stage, the stability of network in the DFAA treatments (Mod = 0.479) was stronger than in the CS treatment (Mod = 0.437). As a more clustered network structure has higher avgCC and APL (Bi et al., 2021), the cluster degree of network in the DFAA treatment was lower than in the CS treatment both after DFAA/natural rainfall and at the mature stage.

In general, the above analysis revealed that the DFAA had significant impacts on topsoil microecological environment in summer maize farmland systems, which led to unstable and dispersed structure of network related to P cycling. The network stability could recover after DFAA, to be even more stable at the mature stage compared with CS treatment. Previous research reported that soil microbial community composition became stable and adapted to such moisture condition under frequent wet-dry cycles (Fierer et al., 2003; Evans and Wallenstein, 2012). Our study revealed that the negative effects of DFAA on PSM would inhibit the transformation of insoluble P into soluble P (including AP), which affected P cycling, and also dispersed the network structure. The network stability of DFAA treatments at the mature stage showed a similar trend. However, the cluster degree would not recover after DFAA at the mature stage. It revealed that DFAA could continuously disperse the farmland ecosystem network related to P cycling.

## Conclusion

Our study focused on the responses of PSM mediated P cycling to DFAA in topsoil of summer maize farmland. The results showed that the DFAA has significant effects on PSB and PSF, especially the genera *Pseudomonas*, *Arthrobacter* and *Penicillium*. The PSF has lower resistance but higher resilience than PSB facing to DFAA as the relative abundance of PSF decreased significantly after DFAA but recovered to the initial state at the mature stage. The DFAA mainly had significant effects on the P metabolism gene K21195, which affect protein synthesis and may be toxic to microbial cells. The DFAA had significant impacts on soil physicochemical and microecological environment, which led to unstable and dispersed structure of farmland ecosystem network related to P cycling. The effects of DFAA on P cycling could last until the mature stage of summer maize. These results provide an empirical basis for the exploration of DFAA on P cycling at the micro and ecosystem level. Further studies could continue carrying out field experiments on the DFAA with other drought-flood combinations, attempting to apply microbial strains improvement and genetic modification for targeted regulation of P transformation and cycling under DFAA.

## Data Availability Statement

The datasets presented in this study can be found in online repositories. The names of the repository/repositories and accession number(s) can be found below: www.ncbi.nlm.nih.gov/, PRJNA761842.

## Author Contributions

WB, BW, and DY contributed to the conception and design of the study. HW confirmed and guided the study. WB, MW, and SY carried out the experiments. TL and WC provided experimental support. WB performed the statistical analysis and wrote the first draft of the manuscript. MW, LJ, TL, and WC wrote sections of the manuscript. All authors contributed to manuscript revision, read, and approved the submitted version.

## Conflict of Interest

The authors declare that the research was conducted in the absence of any commercial or financial relationships that could be construed as a potential conflict of interest.

## Publisher’s Note

All claims expressed in this article are solely those of the authors and do not necessarily represent those of their affiliated organizations, or those of the publisher, the editors and the reviewers. Any product that may be evaluated in this article, or claim that may be made by its manufacturer, is not guaranteed or endorsed by the publisher.
