# Supplementary material for: Responses of Phosphate-Solubilizing Microorganisms Mediated Phosphorus Cycling to Drought-Flood Abrupt Alternation in Summer Maize Field Soil
Source: Front Microbiol. 2022 Jan 13;12:768921. doi: 10.3389/fmicb.2021.768921 (PMC8802831; doi:10.3389/fmicb.2021.768921)
Supplement: Supplementary file 1 [file Data_Sheet_1.docx]

Supplementary Material

The supplementary material supplies the determination method for drought-flood abrupt alternation (DFAA) evaluation (S1), detailed method description for soil phosphorus (P) analysis (S2), determination standard of DFAA in the Northern Anhui Plain (Table S1), experimental setting (Table S2), part of analysis results of phosphate-solubilizing bacteria (Fig. S1) and phosphate-solubilizing fungi (Fig. S2).

# S1 Determination method for DFAA

The determination method of DFAA events considers both the meteorological and agricultural indicators. The DFAA level on daily scale can be evaluated as follows:

${DFAA}_{L}=\left\{ \begin{aligned} \sum_{j-i}^{j} P_{d,l1}=0, W_{d,l1,m}=\frac{\theta_{d,l1,m}}{F_{c}}\times100 \\ \sum_{j}^{j+n-1} P_{f,l2} \end{aligned} \right.$ (1)

Where, ${DFAA}_{L}$ is the level of the DFAA event;$i$ is the duration (days) of drought period; $j$ is the day begins to rain; $P_{d,l1}$ is the amount of available precipitation; $W_{d,l1,m}$ is the relative soil moisture on day $m$ (%)，$j-i\leq m<j$ is the range of day$m$, which can determine the drought level $l1$; $\theta_{d,l1,m}$ is the soil water content on day$m$(%); $n$ is consecutive rainy days;$P_{f,l2}$ is the total amount of precipitation from day $j$to day$j+n-1$, the flood level $l2$can be determined compared with the precipitation threshold. The DFAA level${DFAA}_{L}$ is determined by the drought level $l1$and the flood level $l2$. The beginning day of the drought period in DFAA event is $j-i$, the beginning day of the flood period in DFAA event is $j$, the drought duration is $i$days, the consecutive rainy days is $n$.

The drought level $l1$is determined by the Standard of Classification for Drought Severity (SL424-2008, Table S1) and the Classification of Meteorological Drought (GB/T 20481-2006). The meteorological drought level is generally assessed by the relative soil moisture of 10-20 cm or 0-40 cm depth (Table S1, Eq. 2).

$W=\frac{\theta}{F_{c}}\times100$ (2)

Where, $W$ is the relative soil moisture (%); $\theta$is the soil water content (%); $F_{c}$is the soil field capacity (%).

The flood level $l2$is determined by the amount of precipitation within 5 days after drought. The classification of flood levels in the Northern Anhui Plain is shown in Table S1.

# S2 Detecting method for soil phosphorus

The AP is measured by the colorimetry method. Orthophosphate reacts with ammonium molybdate to form phosphomolybdic heteropoly acid complex [H_3_P(Mo_3_O_10_)] in acidic environment, ascorbic acid is used to reduce it to form blue complex in the presence of antimony reagent, then for the colorimetric analysis. The specific detecting process can be concluded as: i) pipette the blank solution and the test solution 2 mL to 10 mL in a 50 mL volumetric flask; ii) add water to 15 mL to 20 mL; iii) add 1 drop of dinitrophenol indicator, adjust to yellow with sodium hydroxide solution of 2 mol L^–1^; iv) adjust the pH with sulfuric acid solution of 0.5 mol L^–1^, until the solution turning slightly yellow; v) 5.0 mL molybdenum anti-chromogenic agent, dilute to the mark with water, shake well, place at room temperature above 20 °C for 30 min; and vi) obtain the TP concentration in the blank solution and test solution by colorimetric analysis with 700 nm wavelength on a spectrophotometer.

For soil TP, the blank solution and soil solution to be measured (test solution) should first be prepared. The following steps are: i) pipette the blank solution and the test solution 2 mL to 10 mL (with phosphorus concentration of 5 μg mL^–1^ to 25 μg mL^–1^) in a 50 mL volumetric flask; ii) add water to 15 mL to 20 mL; iii) add 1 drop of dinitrophenol indicator, then adjust the pH with sodium hydroxide and sulfuric acid solution until the solution is slightly in yellow; iv) accurately add 5.0 mL molybdenum anti-chromogenic agent, dilute to the mark with water, shake well, place at room temperature above 20 °C for 30 min, the solution turns blue; v) colorimetric analysis with 700 nm wavelength on a spectrophotometer. The instrument zero point was adjusted by a standard solution of 0.00 μg mL^–1^; and vi) obtain the TP concentration in the blank solution and test solution by the standard curve line.


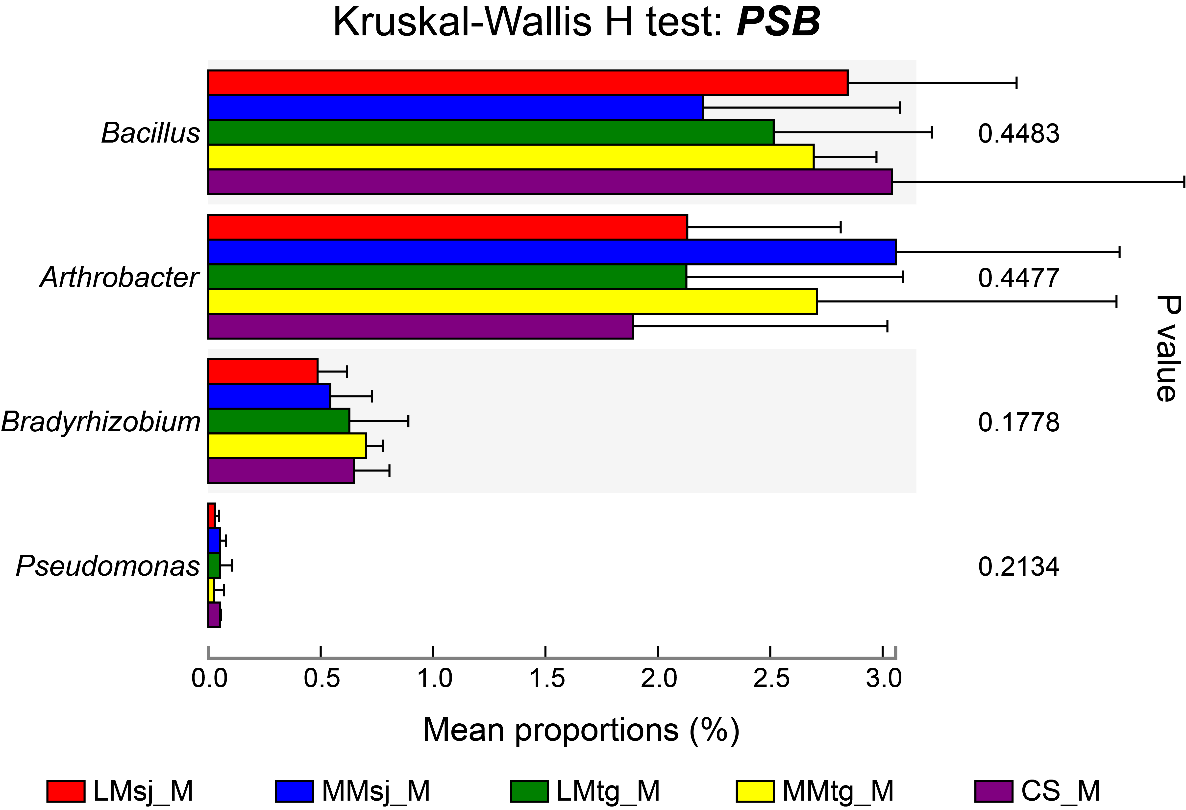


**Supplementary Figure S1.**The Kruskal-Wallis H test bar plot of phosphate-solubilizing bacteria at the mature stage.


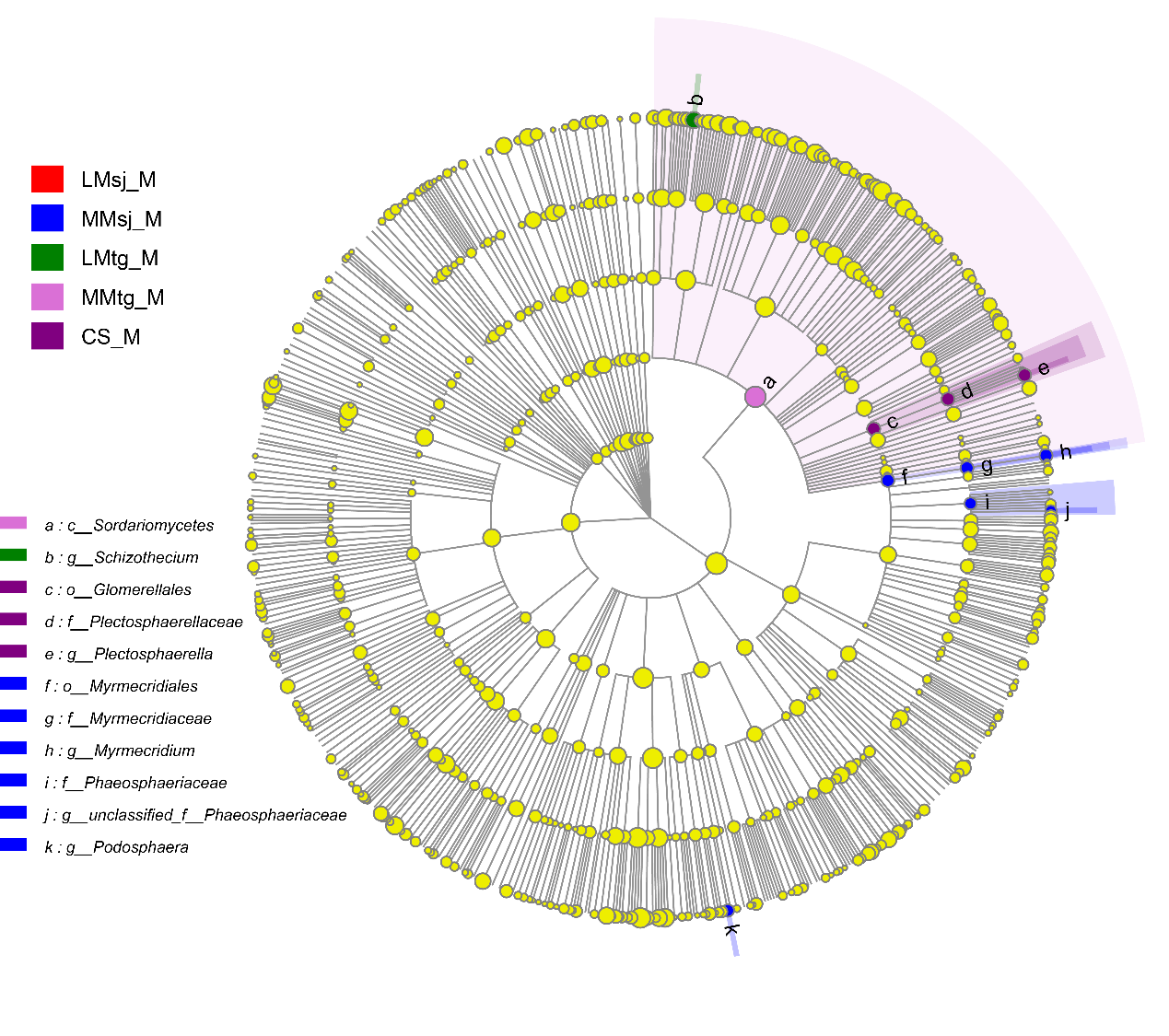


**Supplementary Figure S2.**The LEFSe cladogram of fungal communities (containing phosphate-solubilizing fungi) at the mature stage.

**Supplementary Table S1.** Determination standard of drought-flood abrupt alternation.

| **Drought Level** | | | | | | | **Flood Level** | | | |
| --- | --- | --- | --- | --- | --- | --- | --- | --- | --- | --- |
| Season | **Light** | | **Moderate** | | **Severe** | | CRD (d) | **Light** | **Moderate** | **Severe** |
|  | CnRD (d) | *W* | CnRD (d) | *W* | CnRD (d) | *W* |  | Pre (mm) | Pre (mm) | Pre (mm) |
| Spring (Mar. to May) | 15–30 | 50% <*W* ≤ 60% | 31–50 | 40% <*W*≤ 50% | >50 | *W* ≤ 40% | 1 | 90 | 110 | 135 |
| Summer (Jun. to Aug.) | 10–20 |  | 21–30 |  | >30 |  | 2 | 110 | 140 | 170 |
| Autumn (Sept. to Nov.) | 15–30 |  | 31–50 |  | >50 |  | 3 | 130 | 170 | 210 |
| Winter (Dec. to Feb.) | 20–30 |  | 31–60 |  | >60 |  | 4 | 150 | 190 | 240 |
|  |  |  |  |  |  |  | 5 | 170 | 220 | 280 |

Note: CnRD, continuous rainless days; *W*, relative soil moisture; CRD, continuous rainy days; Pre, the amount of precipitation.

**SupplementaryTable S2.** Settings of drought and flood conditions in different experimental treatments.

| **Treatment** | **Drought set** | | |  | **Flood set** | | | **Maize growing period** |
| --- | --- | --- | --- | --- | --- | --- | --- | --- |
|  | **Soil water content (%)** | **Drought duration (days)** | **Drought level** |  | **Rainfall (mm)** | **Rainfall date** | **Flood level** |  |
| LMsj | 18 | 25 | light |  | 130 | 15 July, 2019 | moderate | seeding-jointing stage |
| MMsj | 15 | 29 | moderate |  | 130 | 19 July, 2019 | moderate | seeding-jointing stage |
| LMtg | 18 | 24 | light |  | 130 | 15 Aug. 2019 | moderate | tasseling-grain filling stage |
| MMg | 15 | 26 | moderate |  | 130 | 17 Aug., 2019 | moderate | tasseling-grain filling stage |
| CS2 | - | - | - |  | 109.2 | 10 Aug., 2019 | - | tasseling-grain filling stage |
